# How to reform western care payment systems according to physicians, policy makers, healthcare executives and researchers: a discrete choice experiment

**DOI:** 10.1186/s12913-015-0847-7

**Published:** 2015-05-06

**Authors:** Roselinde Kessels, Pieter Van Herck, Eline Dancet, Lieven Annemans, Walter Sermeus

**Affiliations:** Faculty of Applied Economics, Department of Economics & StatUa Center for Statistics, University of Antwerp, Prinsstraat 13, B-2000 Antwerpen, Belgium; Department of Public Health and Primary Care, Catholic University of Leuven, Kapucijnenvoer 35, B-3000 Leuven, Belgium; Academic Medical Center, Women’s and Children’s Hospital, Center for Reproductive Medicine, University of Amsterdam, Meibergdreef 9, 1105 AZ Amsterdam, The Netherlands; Leuven University Fertility Center, Leuven University Hospitals, Herestraat 49, B-3000 Leuven, Belgium; Department of Public Health, I-CHER, Ghent University, De Pintelaan 185, B-9000 Ghent, Belgium

**Keywords:** Healthcare payment systems, Healthcare performance objectives, Physician incentive structures, Health policy reform, Discrete choice experiment

## Abstract

**Background:**

Many developed countries are reforming healthcare payment systems in order to limit costs and improve clinical outcomes. Knowledge on how different groups of professional stakeholders trade off the merits and downsides of healthcare payment systems is limited.

**Methods:**

Using a discrete choice experiment we asked a sample of physicians, policy makers, healthcare executives and researchers from Canada, Europe, Oceania, and the United States to choose between profiles of hypothetical outcomes on eleven healthcare performance objectives which may arise from a healthcare payment system reform. We used a Bayesian D-optimal design with partial profiles, which enables studying a large number of attributes, i.e. the eleven performance objectives, in the experiment.

**Results:**

Our findings suggest that (a) moving from current payment systems to a value-based system is supported by physicians, despite an income trade-off, if effectiveness and long term cost containment improve. (b) Physicians would gain in terms of overall objective fulfillment in Eastern Europe and the US, but not in Canada, Oceania and Western Europe. Finally, (c) such payment reform more closely aligns the overall fulfillment of objectives between stakeholders such as physicians versus healthcare executives.

**Conclusions:**

Although the findings should be interpreted with caution due to the potential selection effects of participants, it seems that the value driven nature of newly proposed and/or introduced care payment reforms is more closely aligned with what stakeholders favor in some health systems, but not in others. Future studies, including the use of random samples, should examine the contextual factors that explain such differences in values and buy-in.

**JEL classification:**

C90, C99, E61, I11, I18, O57

## Background

Policy makers of many developed countries are trying to strengthen the long term sustainability of healthcare by reforming healthcare payment systems. They want to replace or enhance salary and fee for service (FFS) payment systems by other incentive structures, such as pay for performance, shared savings, partial capitation, and bundled payment [1]. These incentives aim to reconcile a broader spectrum of healthcare objectives varying from quality, cost and equity to patient centeredness and coordination of care.

The literature to date consists of about 130 effect studies of healthcare payment reform, the majority of them covering the US (50%) and the UK (45%). The remaining studies are spread across Australia, Germany, the Netherlands, Spain and Italy. Studies in Canada and Eastern Europe are largely lacking. Results show that, although reforms seem promising in the long term, short term effects have been disappointing [2-4]. This could partially be explained by growing pains, practical or technical difficulties and the need for short term investments to make the new systems work [5-9]. Nevertheless, more fundamental impediments could be at play. Enthusiasm from physicians and healthcare organizations is lacking as, in contrast to FFS payment systems, new healthcare payment systems require taking financial risks [10,11].

Physicians and healthcare executives are unlikely to engage in a new healthcare payment system if they are not in line with what they value. Knowledge on how different groups of professional stakeholders trade off the merits and downsides of healthcare payment systems is limited. Are the new incentive structures a better match with key priorities of healthcare providers, physicians in particular, even if financial security is at play? Will priorities of physicians, policy makers and healthcare executives converge or diverge as a consequence of the new payment systems? And are the answers to these questions the same across geographical areas with a different health system and context? For each of these questions, we need in-depth knowledge of the values and trade-offs associated with healthcare objectives.

In this paper, we describe the design and analysis results of a discrete choice experiment (DCE) that we performed to examine how improvements, deteriorations and status quo outcomes in healthcare performance objectives due to a payment system reform are traded off by physicians, policy makers, healthcare executives and researchers from Canada, Eastern Europe, Oceania, the US and Western Europe. The DCE is part of a larger study that also includes the rating study of Van Herck et al. [12] in which the same stakeholders directly stated preference ratings for seven healthcare payment systems. The DCE approach is, however, indirect, measuring stakeholder preferences for payment reform outcomes on eleven health system performance objectives. We use the analysis results of the DCE to compare goal fulfillment and stakeholder alignment between current and newly proposed payment structures.

## Methods

The DCE method is a survey technique with a growing use in healthcare to quantify people’s preferences by observing their stated choices in a number of hypothetical scenarios, called choice sets [13-15]. Each choice set consists of two or more competing options, out of which respondents have to indicate the option they like better. The options are also called profiles and are defined in terms of a specified set of attributes or dimensions that differ in a number of levels. The data from a DCE allow the assessment of the relative importance of each attribute in the total value of each of the profiles under study.

Conducting a DCE involves the following steps: (i) identification of the attributes and attribute levels, (ii) experimental design of the choice sets, (iii) questionnaire development, (iv) study sample and (v) data analysis. We discuss these steps in turn.

### Identification of the attributes and attribute levels

As attributes for the DCE, we carefully selected eleven health system performance objectives or domains shown in Table 1. For each objective, we specified three possible outcomes as levels, namely a ‘positive’, ‘negative’ and ‘no change’ outcome.

**Table 1:** **Healthcare system performance objectives or domains considered to be relevant to assess care payment system effects**

**Performance objective**	**Definition**
**1. Clinical effectiveness and patient safety**	The degree to which the level of health gain is maximized and harm to patients is minimized as a consequence of care. This domain refers to the effect of the payment scheme, and its sustainability, on patient outcome in a broad sense (life expectancy, relief of pain, functional capacity, etc.).
**2. Best practice service use**	The degree to which services are provided based on scientific knowledge to all who could benefit (avoiding underuse) and are refrained from being provided to those not likely to benefit (avoiding overuse). This implies that (1) patients do not receive care that cannot help them and/or the risks of which outweigh the benefits and (2) patients reliably receive care where the known benefits outweigh the risks.
**3. Care equity**	The degree to which care and its optimal outcome are delivered and attained for all people, without variation based on patient characteristics (such as gender, age, ethnicity, geographical location and socioeconomic status), unless there is a valid clinical rationale.
**4. Care coordination, teamwork and continuity**	The degree to which provider contributions are well integrated to optimize the delivery of care by the same healthcare provider throughout the course of care, with appropriate and timely communication, referral and collaboration between providers (both within and between provider organizations).
**5. Patient centeredness**	The degree to which care is respectful of and responsive to individual patient preferences and values, ensuring that patient preferences and values guide major clinical decisions.
**6. Timeliness**	The degree to which waits and delays are avoided.
**7. Short term cost containment and budget safety**	The degree to which expenditure of financial resources is contained in short term. Short term expenditure may not only be due to cost of care (including potential waste), but also due to investment in system organization (e.g. cost of implementation).
**8. Long term cost containment and budget safety**	The degree to which expenditure of financial resources is contained in long term. Long term expenditure may not only be due to cost of care (including potential waste), but also due to maintenance of system organization (e.g. cost of measuring and updating).
**9. Provider wellness**	The degree to which provider wellness is sustained, improves or deteriorates, as affected by job satisfaction, income (in)security, workload, autonomy and respect of professional values.
**10. Innovation**	The degree to which innovation of care, at the clinical treatment and/or organizational system level, is encouraged. This includes the strategy and investment focus of the provider (e.g. on quality vs. quantity).
**11. Gaming the system**	The degree to which providers consciously or unconsciously manipulate the system to increase personal financial gain. Gaming includes both data manipulation and/or patient selection (shifting care for high expenditure patients to other providers or providing less than appropriate care).

To select the health system performance domains for study in the DCE, we consulted the literature as well as two expert panels. We interviewed 46 representatives of the stakeholder groups of interest to gauge their opinion about care payment systems and their outcomes [16]. Using a broadly exploratory approach, we identified 25 potentially relevant health system performance domains. Based on literature review, we regrouped the performance domains and reduced them from 25 to 12. We then asked 23 international care payment experts to rate each of the 12 health system performance domains on a 5-point scale in terms of their importance in health policy decision making about care payment systems. The importance rankings of the 12 performance domains led us to select 11 performance domains most likely to influence the preferences for care payment outcomes.

### Experimental design of the choice sets

The DCE presented participants with 18 choice sets of two alternative profiles with performance outcomes that payment change in their health system would generate. For each choice set, participants had to indicate the profile they prefer. The profiles concerned hypothetical combinations of ‘positive’, ‘negative’ or ‘no change’ outcomes on the eleven health system performance domains under study. We designed each choice set using a maximum of five domains to reduce the cognitive burden on the respondents. The profiles in such choice sets are called ‘partial profiles’ [17-19].

**Figure 1:**
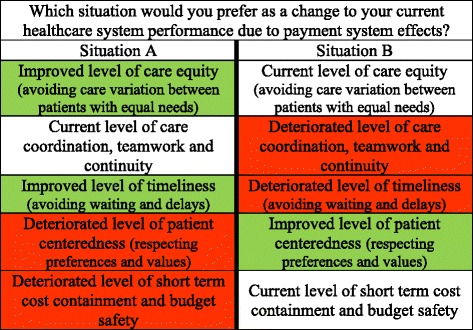
Example of a discrete choice task.

We did not label the partial profiles in terms of specific care payment systems to ensure that only outcome combinations as such determined participants’ preferences and not subjective connotations that specific care payment systems may carry. Coloring highlighted the positive and negative outcomes to facilitate the preference formation process. Figure 1 shows an example of a discrete choice task in which participants had to choose between situation A and situation B.

We created three different surveys by constructing a partial profile design consisting of 54 choice sets and dividing it into three groups of 18 choice sets. The three surveys appear in Appendix A. We submitted the three surveys to groups of three respondents to make sure that each survey was filled out about an equal number of times. Using a design consisting of different surveys results in more precise parameter estimates of the underlying discrete choice model than a design consisting of a single survey [19,20]. As discrete choice model, we used a multinomial logit (MNL) model, which is common practice in discrete choice design and analysis [21]. The partial profile design in Appendix A is D-optimal for the MNL model, meaning that it guarantees precise parameter estimates [22].

Each choice set of the partial profile design in Appendix A varies the levels of at most five attributes and ignores the remaining attributes, which are assumed constant at any possible level indicated by ‘*’ and ‘§’ signs. These constant attributes differ from choice set to choice set. We determined the constant attributes with the ‘*’ sign using the attribute balance approach that attempts to hold each attribute constant in an equal number of choice sets and to pair constant attributes an equal number of times [18,19]. That is why over all 54 choice sets in the design, each attribute is constant in either 29 or 30 choice sets, not taking into account the constant attributes with the ‘§’ sign. The latter ones are added to make the design D-optimal, resulting in a better estimation performance.

The D-optimal partial profile design takes into account prior beliefs about the respondents’ preferences. It generally holds that a ‘positive’ outcome in a performance domain is preferred to a ‘no change’ outcome, which, in turn, is preferred to a ‘negative’ outcome. In addition, according to prospect theory which states that people are loss averse, avoiding a negative outcome is likely more dominant in preference formation compared to trading off a neutral versus a positive outcome [23]. We also ranked the performance domains in order of expected importance and expressed our uncertainty regarding the a priori orderings of the performance domains and the performance outcomes in a multivariate normal prior distribution. In Appendix B, we describe the multivariate normal prior distribution used to optimize the design of the DCE. The design that maximizes the information content of the DCE (as measured by the log-determinant of the information matrix; see [22]), when averaged over a given prior distribution, is called a Bayesian D-optimal design. The Bayesian D-optimal design approach is increasingly considered state-of-the-art for constructing DCEs [22,24-26]. One major benefit of Bayesian D-optimal designs is that, using a proper prior distribution, they avoid choice sets in which one profile is completely dominating the other profile(s) on every attribute [27].

### Questionnaire development

The actual questionnaire consisted of two parts. In the first part, we asked respondents a number of background questions such as their age, seniority, gender, stakeholder role and domain of expertise. Also, we questioned respondents on the geographical area they work in, the healthcare payment systems in use, their specialty or degree of medicine and the care practice setting. In the second part, we performed the DCE by presenting respondents with 18 choice sets of two performance outcome profiles. The questionnaire language was English and data were collected online over the year 2011.

The medical ethics committee of the University Hospitals Leuven - UZ Leuven, Belgium, waived the need for a formal approval of the study, due to the non-interventional nature and because the study is not directed at patients. We obtained a written informed consent from all participants.

### Study sample

The study sample consisted of physicians, policy makers, healthcare executives and individual healthcare payment researchers across Canada, Eastern Europe, Oceania, the US and Western Europe, but was not designed to be representative of each of these four stakeholder groups and five geographical areas. To reach physicians, policy makers and healthcare executives, we asked 48 international professional healthcare societies within the field of medicine, health policy, care management and public health research to invite their members to participate in the study. To reach researchers, we sent 2,051 authors of peer-reviewed papers on the topic of care payment systems an invitation by email to participate. The healthcare societies could make use of various communication channels to invite members to participate, including direct emailing, newsletters and/or websites. Some societies applied randomized sampling to select members, whereas others invited all their members. In addition, membership to a society consists of sub-societies (e.g. at the national level within Europe), organizations (e.g. hospitals), and/or individual members. The two former types could make use of a cascade invitation procedure to support involvement. To minimize the workload and to standardize the process, we provided the societies with preformatted letters containing the web survey links.

A total of 547 stakeholders participated in the study. Their characteristics are shown in Table 2. Respondents are predominantly male (69%) with a mean age of 50 years and a mean seniority of 23 years. They assume the following stakeholder role(s): physicians (67%), policy makers (22%), healthcare executives (34%) and researchers (30%). The most commonly cited domain of expertise is medicine (69%) and among the geographical areas the US is most represented (37%). Also, a large part of the physicians work in teaching hospitals (34%). Stakeholders evaluated 8,544 of a total of 9,846 choice sets shown (computed as 547*18 choice sets), which corresponds to a completion rate of 87%.

**Table 2:** **Characteristics of the 547 respondents in the study**

**Characteristic**	**Value**
Age	50 ± 11 years^*^
Seniority	23 ± 11 years^*^
Female sex	31%
*Stakeholder role, based on work content°*	
Physician	67%
Policy maker	22%
Healthcare executive	34%
Researcher	30%
*Domain of expertise, self-rated°*	
Medicine	69%
Nursing	3%
Allied health	6%
Policy	17%
Executive management	17%
Financial management	8%
Public health	10%
Quality of care	17%
Health economics	13%
Psychology	2%
Social sciences	3%
Human resource management	4%
Law	5%
Ethics	4%
Insurance	5%
Pharmacy	3%
*Geographical area*	
Canada	10%
Eastern Europe	9%
Western Europe	25%
Oceania	18%
United States of America	37%
*Practice setting* ^*§*^ *°*	
Solo primary care	13%
Group primary care	19%
Non-teaching hospital	8%
Teaching hospital	34%
*Care payment system in use to pay physicians°*	
Salary	67%
Fee for service	60%
Episode-based	6%
Capitation	16%
Quality bonus or adjustment	16%
Evidence informed case rate	2%
Never event non-reimbursement/warranty	1%

^*^The ± values are means ± SD.

°Respondents could select more than one response category.

^§^These characteristics pertain to physicians only.

### Data analysis

We estimated a MNL model describing the overall preferences for care payment reform outcomes in the eleven performance domains, taken into account the impact of stakeholder role and geographical area and adjusting for age, seniority, gender and care payment systems in use. Respondents’ propensity to opt for a profile is the model’s dependent variable. The eleven performance domains are the main independent variables which are assumed categorical, having the ‘positive’, ‘negative’ and ‘no change’ payment outcomes as levels.

The MNL model relies on a utility function that is the sum of the marginal utilities of the attributes’ main and interaction effects under study [28]. The interactions involve combinations of attributes and respondent subgroup variables to investigate the heterogeneity in preferences between respondent subgroups. We estimated the model effects or parameters using a maximum likelihood approach. We computed the overall significance of the attributes using likelihood ratio (LR) tests and measured their relative importance by –log(p-value of the LR test). Because absolute values of the marginal utilities have no direct interpretation, we expressed all parameters relative to a reference parameter or ‘golden standard’. As a reference parameter, we chose the parameter attached to a deterioration in ‘effectiveness and patient safety’, because a deterioration in this domain has, by far, the highest (negative) impact on respondents’ choices (see Results). We standardized all parameters with respect to the absolute value of the reference parameter, so that the parameter values are to be interpreted with respect to the reference value of -1, with the sign indicating a positive (+) or negative (-) impact on choice. We carried out the entire data analysis using the Choice Modeling platform in the statistical software package JMP 11.

## Results

### Performance objectives defining the choice for a healthcare payment system

**Figure 2:**
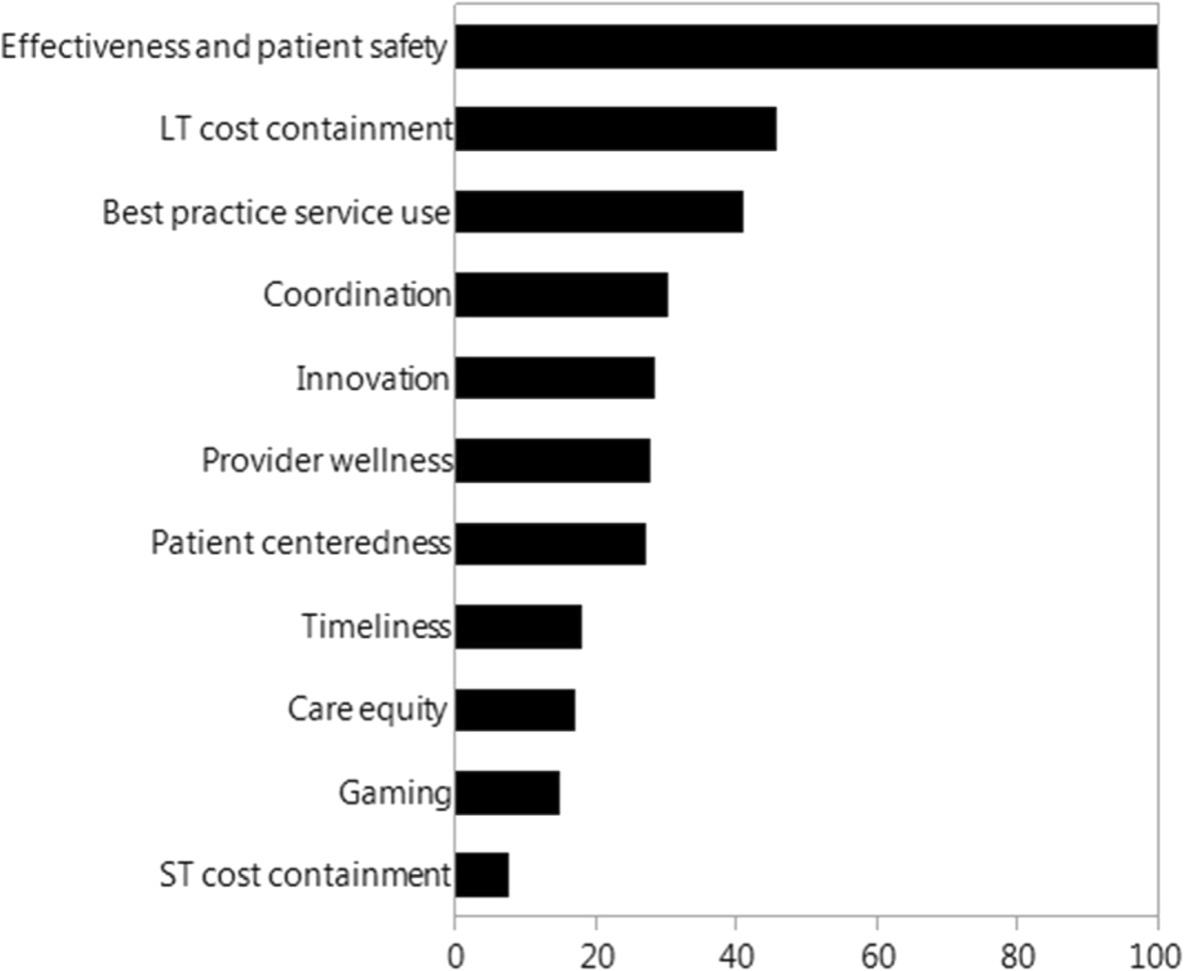
Importance of the eleven healthcare performance objectives or domains in the MNL model relative to the most important objective ‘effectiveness and patient safety’.

All eleven healthcare performance objectives or domains have a significant impact on stakeholders’ choices (p < 0.0001) after adjustment for age, seniority, gender, care payment systems in use, stakeholder role and geographical area. Figure 2 ranks the performance domains in order of importance. The importance of a domain is expressed relatively to the most important domain ‘effectiveness and patient safety’, the importance of which is set to 100. The domain next in line is ‘long term cost containment’ and is only about half as important as ‘effectiveness and patient safety’. ‘Provider wellness’, which includes physicians’ income, a key element to most health economic models of care payment, takes up a sixth position. The domains ‘timeliness’, ‘care equity’, ‘gaming’ and ‘short term cost containment’ are least important in setting objectives for care payment reform.

### The importance of care payment reform outcomes in each performance domain

**Figure 3:**
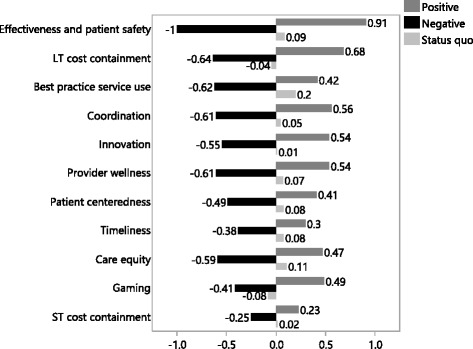
Marginal utility values (main effects) of the positive, negative and status quo outcomes related to the eleven healthcare performance objectives.

Figure 3 shows the marginal utility values attached to the positive, negative and status quo outcomes in each performance domain. These marginal utility values are significant main effects (p < 0.0001) expressed relatively to the golden standard of no harm to effectiveness, the main effect of which is set to -1. The values do not fully align with the importance ranking of the performance domains shown in Figure 2, which is due to the presence of a number of significant interaction effects. Especially for the less important domains (such as ‘timeliness’ and ‘care equity’), interactions co-determine the impact on total utility (see Impact of stakeholder role and Impact of geographical area).

As expected, avoiding unintended consequences (negative payment outcomes) is generally of higher importance than intended ‘improvement’ consequences (positive outcomes). Only for ‘long term cost containment’ and ‘gaming’, improvement is somewhat more important than avoiding deterioration. The status quo outcomes in the performance domains influence the stakeholders’ choices barely, mostly in a positive way. Only the status quo outcomes of ‘long term cost containment’ and ‘gaming’ have a slightly negative impact on stakeholders’ choices.

### Impact of stakeholder role

We compare the choices of each of the four stakeholder groups to those of the other three stakeholder groups. We first examine the differences in marginal utility values of care payment reform outcomes between physicians and non-physicians. We then proceed with a discussion on the choices of policy makers, healthcare executives and researchers.

**Figure 4:**
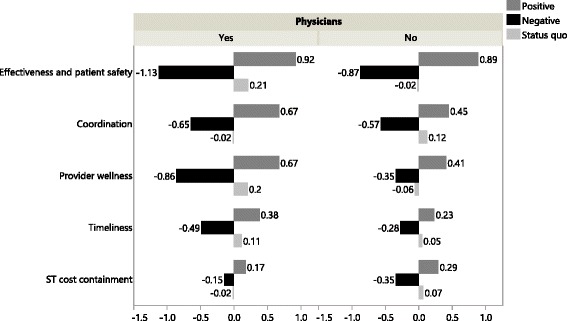
Marginal utility values of the positive, negative and status quo outcomes showing significant differences in preference evaluation between physicians and non-physicians.

As shown in Figure 4, physicians differ from non-physicians with respect to their preferences for five of the eleven performance objectives. More specifically, physicians attach more importance to ‘effectiveness and patient safety’ (p = 0.0046), ‘coordination’ (p = 0.0097), ‘provider wellness’ (p < 0.0001) and ‘timeliness’ (p = 0.0044) than non-physicians. For ‘short term cost containment’, the opposite is true (p = 0.0057).

Concerning ‘effectiveness and patient safety’, avoiding harm affects physicians’ choices 0.30 times more (computed as 1.13/0.87 – 1) than non-physicians’ choices. On the other hand, physicians and non-physicians value an improvement in this performance domain through payment reform to about the same extent. Concerning ‘coordination’, physicians value reform that avoids deterioration and provides improvement more than non-physicians (0.14 and 0.49 times more, respectively). This is also the case for ‘provider wellness’ and ‘timeliness’, but the differences are more outspoken (1.46 and 0.63 times more for ‘provider wellness’, and 0.75 and 0.65 times more for ‘timeliness’). The opposite is true for ‘short term cost containment’. For this performance domain, physicians prefer avoiding deterioration and enhancing improvement less than non-physicians (0.57 and 0.41 times less).

Investigating policy makers as a stakeholder group, we observe only a significant difference in preference evaluation for ‘gaming’ compared to non-policy makers (p = 0.0002). Policy makers attach less importance to avoiding deterioration/expansion in gaming (0.16 times less) and more importance to improving/reducing gaming (0.72 times more).

Considering healthcare executives, we observe only a significant difference in preferences for ‘provider wellness’ compared to non-healthcare executives (p < 0.0001). Healthcare executives favor avoiding deterioration and enhancing improvement more than non-healthcare executives (0.57 and 0.43 times more).

The last stakeholder group concerns the researchers with an expertise in care payment reform. Researchers pay more attention to ‘effectiveness and patient safety’ (p = 0.0021) and ‘long term cost containment’ (p = 0.0002) than non-researchers. They favor avoiding deterioration and enhancing improvement more than non-researchers (0.30 and 0.26 times more for ‘effectiveness and patient safety’, and 0.67 and 0.39 times more for ‘long term cost containment’).

### Impact of geographical area

**Figure 5:**
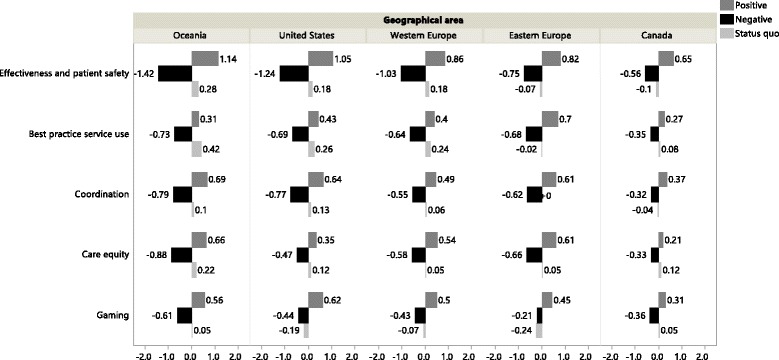
Marginal utility values of the positive, negative and status quo outcomes showing significant differences in preference evaluation between geographical areas.

Figure 5 shows the significant geographical differences in the valuation of five of the eleven performance domains: ‘effectiveness and patient safety’ (p < 0.0001), ‘best practice service use’ (p = 0.0004), ‘coordination’ (p = 0.0096), ‘care equity’ (p = 0.0056) and ‘gaming’ (p = 0.0402).

Overall, stakeholders from Oceania pay the most importance to the effects of care payment reform in the five performance domains, while stakeholders from Canada pay the least importance. The importance values of stakeholders from the US and Western and Eastern Europe range in between. The preferences of stakeholders from the US best match with those of stakeholders from Oceania. Both are by far most concerned about the effects of a change in ‘effectiveness and patient safety’. Together with stakeholders from Western Europe, they favor avoiding deterioration more than improvement in this domain. This is in contrast with stakeholders from Eastern Europe and Canada, who attach more importance to improvement in ‘effectiveness and patient safety’ than to avoiding deterioration.

### Impact of physicians’ payment systems currently in use

We compare stakeholders’ choices based on the current payment systems in use to pay physicians (see Table 2 for the different payment forms). Because respondents could select more than one payment form, it is not surprising that we found no significant differences in the evaluation of the care payment reform outcomes between most payment forms in use, including salary, fee for service, episode-based payment, capitation, evidence informed case rates and never event non-reimbursement or warranty. There is, however, a significant effect from the use of a quality bonus or adjustment. Using this payment form, stakeholders pay less importance to the outcome effects in ‘provider wellness’ compared to not using it (0.41, 0.35 and 0.73 times less for avoiding deterioration, improvement and preserving status quo).

### Comparison of goal fulfillment and alignment between current and newly proposed payment structures

Newly proposed payment structures differ from current payment structures in the sense that they include a widening of scope of payment through bundled or global payment, a strengthening of primary care by means of partial capitation and a risk sharing component in the form of episode-based payment or capitation. These elements aim to improve coordination, innovation and long term cost containment.

A second difference with current payment structures is the inclusion of evidence based process and outcome criteria in payment to improve effectiveness and patient safety, best practice service use and long term cost containment by ways of pay for performance, shared savings, evidence informed case rates, and/or warranty use.

In newly proposed payment structures, we also take unintended consequences into account, including a deterioration of gaming [29] and limited neglect of untargeted quality aspects, yet not to an extent that the gain in effectiveness would be undone [30]. We let care equity at status quo, because findings for this objective are inconsistent [31].

Furthermore, a reduced degree of financial security as part of provider wellness is inherent to risk sharing. Reforms also increase short term costs due to transaction costs.

We calculated the relative difference in total utility between the newly proposed payment system and the current payment system for each stakeholder group and each geographical area. To obtain the total utility for the current payment system, we added up the marginal utilities attached to the status quo outcomes for all eleven performance domains. The newly proposed payment system is characterized by improvements in ‘effectiveness and patient safety’, ‘long term cost containment’, ‘best practice service use’, ‘coordination’ and ‘innovation’, by unintended deterioration in ‘provider wellness’, ‘patient centeredness’, ‘timeliness’, ‘gaming’ and ‘short term cost containment’ and by a status quo in ‘care equity’.

Table 3 describes the payment reform impact on total utility for each stakeholder group and each geographical area. Comparing the total utilities between the current and newly proposed payment system using the relative difference between these utilities shows that the new payment system agrees much more with stakeholder preferences than the current payment system for almost all stakeholder groups, except for physicians from Canada, Oceania and Western Europe and healthcare executives from Oceania who would need to concede a trade-off in their utilities. Moreover, one expects that, in order of importance, healthcare executives and researchers from Eastern Europe and policy makers from Canada derive most utility from the new payment system. From a geographical perspective, Eastern Europe clearly has most to gain in total utility after a payment reform.

**Table 3:** **Predicted total utility by stakeholder group, before and after proposed payment reform**

**Geographical area**	**Stakeholder group**	**Total utility of status quo**	**Total utility after proposed payment reform**	**Relative difference** ^*^
Canada	Physician	0.61	0.48	−0.21
	Policy maker	−0.04	0.82	21.50
	Healthcare executive	0.37	0.48	0.30
	Researcher	0.42	1.18	1.81
Oceania	Physician	1.57	1.18	−0.25
	Policy maker	0.92	1.52	0.65
	Healthcare executive	1.33	1.18	−0.11
	Researcher	1.38	1.88	0.36
Eastern Europe	Physician	0.22	1.40	5.36
	Policy maker	−0.43	1.74	4.05
	Healthcare executive	−0.02	1.40	70.00
	Researcher	0.03	2.10	69.00
Western Europe	Physician	0.96	0.80	−0.17
	Policy maker	0.31	1.14	2.68
	Healthcare executive	0.72	0.80	0.11
	Researcher	0.77	1.50	0.95
United States	Physician	1.00	1.23	0.23
	Policy maker	0.35	1.57	3.49
	Healthcare executive	0.76	1.23	0.62
	Researcher	0.81	1.93	1.38

^*^Relative difference is the difference between the total utility after proposed payment reform and the total utility of the status quo, expressed relatively to the total utility of the status quo.

Finally, we studied the degree of goal alignment between physicians, policy makers, healthcare executives and researchers by comparing differences in total utility between the stakeholder groups in the current and new payment system. We observed that the total utilities between stakeholder groups become more closely aligned in the new payment system, in the sense that the differences in total utility between physicians and policy makers diminish. This result is in contrast with the result of the more direct preference enquiry of Van Herck et al. [12], which revealed that care payment preferences between the stakeholder groups appear to be misaligned. There is thus a discrepancy in preferences that stakeholders directly state and those that are indirectly derived from a DCE.

## Discussion

To our knowledge, this is one of the first studies using a DCE to examine how stakeholder preferences are affected by changes in health system performance objectives or domains and what the implications are for care payment reform. Besides the effect of ‘positive’, ‘negative’ and ‘no change’ outcomes on eleven performance objectives, we also investigated the impact of stakeholder role and geographical area, while adjusting for age, seniority and gender of stakeholders. We report results using choice data from 547 respondents.

We found that all eleven performance objectives have a significant impact on stakeholder preferences, with the impact of ‘effectiveness and patient safety’ standing out. Next in line are ‘long term cost containment’ and ‘best practice service use’, which are only about half as important as ‘effectiveness and patient safety’. Also, our initial assumptions have been mostly confirmed: except for ‘long term cost containment’ and ‘gaming’, unintended consequences (negative outcomes) of payment reform have a stronger impact on respondents’ choices than intended consequences (positive outcomes). One possible explanation why preferences for improvement in ‘long term cost containment’ and avoidance of ‘gaming’ are higher despite any unintended consequences is a high level of dissatisfaction with current performance. All stakeholders seem to crave for an improvement in long term cost containment and better checks on gaming of the system.

Physicians, policy makers, health care executives and researchers have their own priorities, differing further by geographical area. Our study findings seem to match well with the work content of each group. Physicians focus more on doing no harm to effectiveness and patient safety, coordination, provider wellness and timeliness. Policy makers are more concerned about gaming and healthcare executives about financial security. Researchers focus more on long term outcome and cost containment.

Our findings confirm that newly proposed payment structures, aligning care payment with health improvement and long term cost containment, combined with a widening of scope, increase the overall fulfillment of the objectives of most stakeholder groups across geographical areas. This is in line with the formalized models of David Cutler, focusing on social welfare optimization [32]. Value is confirmed to be a better care payment criterion than volume and intensity. However, as a consequence of the trade-off with financial risk and other unintended consequences, the overall goal fulfillment of physicians in Canada, Oceania and Western Europe diminishes. Possible explanations are that Oceania is internally perceived as a high performer already, Canadian physicians focus more on conservative objectives for payment, and in Western Europe one does not feel sufficient pressure yet on cost containment. These hypothetical factors are likely to change during the following years and decades as a consequence of external pressure on health systems. In Eastern Europe and the US, this seems to be already the case. In addition, the findings confirm a closer alignment between stakeholder groups as a result of new payment structures, which is opposite to the result of the preference rating study of Van Herck et al. [12], in which stakeholder preferences were directly enquired.

It is noteworthy to observe that all stakeholder groups in all geographical areas attach hardly any value to short term cost consequences of payment reform. In contrast, they value long term cost consequences very highly. Short term cost considerations are very near the tail of the choice criteria. Could it be that stakeholders perceive care payment reform as a long term instead of a short term strategy, because short term gains are not realistic to reach?

Our results should be interpreted with caution because of the following limitations: (1) Findings are based on scenario evaluation and not on real-life observation. We cannot be certain that the choices made in the DCE guide actual decision making. (2) In the DCE we focused on large geographical areas, and not on separate countries or states. In this respect, Eastern Europe is probably the most heterogeneous area in terms of healthcare payment systems, attitude of health policy makers and national income. (3) As healthcare providers among the stakeholders, we focused on physicians only, and did not consider nurses or other medical assistants. (4) A sample size of 547 participants is limited from a non-DCE survey perspective. However, because most participants in the DCE contributed to 18 observations each, a sample size of 547 is considered to be large. The large amount of significant results from this study confirm our reasoning. (5) The sample is most likely representative of motivated, early responders only, who have a good understanding of English.

Based on the study findings, we formulate the following recommendations:

First and foremost, unintended consequences for effectiveness and patient safety should be avoided by means of risk adjustment, evening out ‘treating to the test’ in pay for performance and monitoring of payment consequences.

Second, all stakeholder groups highly value a positive effect of payment reform on effectiveness and patient safety. Policy makers should regularly demonstrate, based on the targets that physicians care about, that payment reform indeed improves effectiveness and patient safety, albeit often in subsequent incremental steps.

Third, we recommend accommodating for provider wellness in any way possible. Many proposed reforms share an inherent reduction of physicians’ financial security. The same seems true for autonomy. Yet, there are payment design formulas that respect autonomy of care, while simultaneously encouraging appropriateness of care whenever possible [33]. Respect for professional values also depends on how, and by whom, a care payment format is specifically designed [34,35].

Finally, we recommend customizing care payment reform based on contextual values, according to the geographical area, which should be examined further in future research.

## Conclusion

To ensure the support of stakeholders, future care payment reform should actively incent effectiveness and patient safety, both in terms of inducing improvement and avoiding harm. Payment system design should increase provider wellness in other ways than offering financial security. As the DCE showed, if policy makers pay sufficient and convincing attention to intended and unintended consequences on the full pallet of healthcare objectives, physicians and healthcare executives may positively trade off part of their financial security. Their end point, however, depends on the health system in which they operate. Priorities should be further customized according to the local context of care. Future research should examine whether our findings can be confirmed at the level of individual countries, using a probability sample. Furthermore, also the perspective of patients and the general public as a whole should be included in future DCEs, since objectives of care relate to the general priorities that citizens hold for their health system.

## Appendix A: Bayesian D-optimal partial profile design consisting of three surveys for the DCE

The design of the DCE involved three surveys of 18 choice sets with two alternative combinations of performance outcomes. The surveys appear in Tables 4, 5 and 6. The choice sets in each survey were presented in a randomized order to the respondents and each survey was filled out by about 180 respondents. The eleven attributes correspond to the eleven performance domains of Table 1. The ‘+’ sign denotes the positive outcome in each performance domain, ‘N’ denotes the no change or neutral outcome, and the ‘–’ sign denotes the negative outcome. Constant attributes that are dropped from the choice sets are indicated by ‘*’ and ‘§’ signs. Each choice set has at least six constant attributes. For choice sets that have more than six constant attributes, the additional constant attributes are indicated by the ‘§’ sign.

**Table 4:** **Survey 1 of the Bayesian D-optimal partial profile design**

**Choice**	**Attributes**										
**set**	**1**	**2**	**3**	**4**	**5**	**6**	**7**	**8**	**9**	**10**	**11**
**1**	*	*	*	*	+	N	+	–	*	*	–
**1**	*	*	*	*	N	–	–	+	*	*	N
**2**	*	*	–	*	*	–	*	+	–	N	*
**2**	*	*	+	*	*	N	*	N	N	–	*
**3**	*	*	–	*	*	+	N	+	N	*	*
**3**	*	*	+	*	*	N	+	–	–	*	*
**4**	*	*	N	+	*	–	*	–	*	+	*
**4**	*	*	–	N	*	+	*	N	*	–	*
**5**	*	–	*	*	§	§	*	*	*	N	+
**5**	*	N	*	*	§	§	*	*	*	+	–
**6**	*	N	*	§	–	*	N	+	*	*	*
**6**	*	–	*	§	N	*	–	–	*	*	*
**7**	*	–	*	*	N	+	*	*	*	–	–
**7**	*	N	*	*	–	N	*	*	*	N	+
**8**	*	N	§	–	–	*	*	*	N	*	*
**8**	*	–	§	N	N	*	*	*	–	*	*
**9**	*	+	N	*	+	*	N	*	*	+	*
**9**	*	N	+	*	N	*	–	*	*	–	*
**10**	N	*	*	§	*	*	+	+	§	*	*
**10**	–	*	*	§	*	*	–	N	§	*	*
**11**	N	*	*	*	*	+	–	–	–	*	*
**11**	+	*	*	*	*	–	+	N	+	*	*
**12**	+	*	*	*	N	*	*	N	N	*	–
**12**	–	*	*	*	+	*	*	–	+	*	N
**13**	N	*	*	*	+	–	*	*	*	N	–
**13**	+	*	*	*	N	N	*	*	*	+	N
**14**	+	*	*	N	*	+	*	*	–	*	N
**14**	–	*	*	–	*	N	*	*	+	*	+
**15**	+	*	*	–	*	*	N	–	*	+	*
**15**	–	*	*	+	*	*	–	+	*	–	*
**16**	–	*	–	*	N	*	+	*	*	*	N
**16**	+	*	+	*	–	*	–	*	*	*	+
**17**	–	+	–	*	*	*	–	*	*	*	+
**17**	+	N	+	*	*	*	N	*	*	*	N
**18**	N	–	+	N	*	*	N	*	*	*	*
**18**	–	N	N	–	*	*	+	*	*	*	*

**Table 5:** **Survey 2 of the Bayesian D-optimal partial profile design**

**Choice**	**Attributes**										
**set**	**1**	**2**	**3**	**4**	**5**	**6**	**7**	**8**	**9**	**10**	**11**
**19**	*	*	*	*	+	§	–	*	–	*	N
**19**	*	*	*	*	N	§	N	*	N	*	–
**20**	*	*	*	*	–	N	§	N	§	*	*
**20**	*	*	*	*	N	–	§	+	§	*	*
**21**	*	*	*	+	–	§	*	*	+	*	–
**21**	*	*	*	–	N	§	*	*	N	*	+
**22**	*	*	*	N	N	*	*	N	*	N	N
**22**	*	*	*	+	+	*	*	+	*	–	–
**23**	*	*	–	N	*	*	*	*	N	+	–
**23**	*	*	+	–	*	*	*	*	+	–	N
**24**	*	*	+	+	*	*	N	*	*	–	N
**24**	*	*	N	N	*	*	–	*	*	+	–
**25**	*	N	*	*	+	*	*	N	+	–	*
**25**	*	–	*	*	–	*	*	+	–	N	*
**26**	*	–	*	*	–	+	+	*	*	*	N
**26**	*	+	*	*	N	–	N	*	*	*	–
**27**	*	–	*	+	*	*	*	–	N	*	+
**27**	*	+	*	–	*	*	*	+	+	*	–
**28**	*	N	§	+	§	*	–	*	*	*	*
**28**	*	–	§	–	§	*	+	*	*	*	*
**29**	*	N	N	*	*	+	*	*	*	–	+
**29**	*	–	–	*	*	N	*	*	*	+	–
**30**	*	–	N	*	*	N	*	+	*	*	+
**30**	*	+	–	*	*	–	*	–	*	*	N
**31**	+	*	§	§	*	*	–	*	*	N	*
**31**	N	*	§	§	*	*	+	*	*	+	*
**32**	N	*	*	*	N	N	–	N	*	*	*
**32**	+	*	*	*	–	+	+	–	*	*	*
**33**	N	N	*	*	*	*	N	*	–	+	*
**33**	+	–	*	*	*	*	+	*	+	N	*
**34**	–	+	*	N	*	*	*	*	–	–	*
**34**	N	N	*	–	*	*	*	*	+	N	*
**35**	N	+	–	§	*	N	*	*	*	*	*
**35**	+	N	N	§	*	+	*	*	*	*	*
**36**	+	–	–	*	–	*	*	*	*	+	*
**36**	–	+	+	*	N	*	*	*	*	–	*

**Table 6:** **Survey 3 of the Bayesian D-optimal partial profile design**

**Choice**	**Attributes**										
**set**	**1**	**2**	**3**	**4**	**5**	**6**	**7**	**8**	**9**	**10**	**11**
**37**	*	*	+	*	*	§	+	§	*	§	*
**37**	*	*	N	*	*	§	–	§	*	§	*
**38**	*	*	+	*	*	*	–	N	–	+	*
**38**	*	*	N	*	*	*	+	–	N	–	*
**39**	*	*	+	§	*	+	*	*	§	+	*
**39**	*	*	–	§	*	–	*	*	§	N	*
**40**	*	*	–	*	N	*	+	*	N	*	+
**40**	*	*	+	*	+	*	N	*	–	*	–
**41**	*	–	*	*	*	N	+	*	N	–	*
**41**	*	N	*	*	*	+	N	*	–	+	*
**42**	*	+	*	+	*	+	*	N	*	*	–
**42**	*	–	*	N	*	–	*	+	*	*	N
**43**	*	N	*	–	–	*	+	N	*	*	*
**43**	*	–	*	+	N	*	N	+	*	*	*
**44**	*	N	N	*	N	*	*	+	–	*	*
**44**	*	+	+	*	+	*	*	N	N	*	*
**45**	–	*	*	*	*	*	*	+	N	N	N
**45**	+	*	*	*	*	*	*	N	+	–	–
**46**	–	*	*	§	*	–	+	*	*	*	+
**46**	+	*	*	§	*	+	–	*	*	*	N
**47**	–	*	§	N	N	*	*	*	N	*	*
**47**	+	*	§	+	+	*	*	*	+	*	*
**48**	–	*	*	N	+	*	*	N	*	N	*
**48**	+	*	*	–	N	*	*	+	*	+	*
**49**	+	*	*	+	–	N	*	*	*	–	*
**49**	N	*	*	N	N	–	*	*	*	+	*
**50**	–	*	+	–	*	+	*	*	*	*	+
**50**	+	*	N	+	*	–	*	*	*	*	N
**51**	+	–	*	*	*	*	*	N	*	–	+
**51**	N	+	*	*	*	*	*	–	*	+	N
**52**	N	–	*	+	*	+	*	*	N	*	*
**52**	–	N	*	N	*	N	*	*	+	*	*
**53**	–	+	+	*	*	*	*	N	*	*	–
**53**	N	–	N	*	*	*	*	–	*	*	+
**54**	–	–	N	*	+	*	*	*	N	*	*
**54**	N	+	+	*	N	*	*	*	–	*	*

## Appendix B: Multivariate normal prior parameter distribution used to construct the Bayesian D-optimal partial profile design for the DCE

To construct the Bayesian D-optimal partial profile design for the DCE shown in Appendix A, we used a multivariate normal prior distribution that reflects the prior beliefs about the unknown parameter values associated with the attribute levels, i.e. the care payment reform outcomes in the eleven performance domains. Based on expert interviews and literature review, we ranked the eleven performance domains in order of importance and specified mean parameter values and variances for the multivariate normal prior distribution.

Table 7 shows the eleven performance domains in order of importance. The more important a domain is believed to be prior to our study, the larger in magnitude the a priori mean utility values specified for the main effects of that domain. Note that the utility values associated with the outcomes in each performance domain sum to zero. This is because we used effects coding for the outcomes which means that we coded the negative outcome as [1 0], the neutral outcome as [0 1] and the positive outcome as [-1 -1]. To indicate that people are loss averse, we chose larger absolute mean values for the negative outcome in each domain than for the positive outcome. Because of the zero-sum constraint, the mean values for the neutral outcome are slightly larger than zero.

**Table 7:** **A priori order of importance of the main effects of the eleven performance domains and conversion into mean utility values and variances for the multivariate normal prior distribution**

**Rank**	**Performance domain**	**Prior mean**			**Prior variance**
		**–**	**N**	**+**	
1	Clinical effectiveness and patient safety	−0.6	0.1	0.5	0.09
2	Best practice service use	−0.4	0.05	0.35	0.06
	Long term cost containment and budget safety	−0.4	0.05	0.35	0.06
3	Gaming the system	−0.35	0.05	0.3	0.04
	Care equity	−0.35	0.05	0.3	0.04
	Care coordination, teamwork and continuity	−0.35	0.05	0.3	0.04
4	Timeliness	−0.3	0.05	0.25	0.02
	Patient centeredness	−0.3	0.05	0.25	0.02
	Innovation	−0.3	0.05	0.25	0.02
	Provider wellness	−0.3	0.05	0.25	0.02
	Short term cost containment and budget safety	−0.3	0.05	0.25	0.02

The mean values are associated with the negative (–), neutral (N) and positive (+) outcomes in each performance domain. Variances around the mean values are the same for all three outcomes in a performance domain and are therefore listed only once.

For the prior variances around the mean utility values, we wanted to preserve the natural rank order of the outcomes in each performance domain. We therefore chose variances such that the standard deviations are smaller than the difference between the mean values for the positive and neutral outcomes in a performance domain. Following a suggestion of Kessels et al. [36], we also specified negative covariances between the prior utility values associated with each performance domain. We computed these covariances using a correlation coefficient of -0.5, such that for the performance domains in each of the four ranks the covariances equal -0.05, -0.03, -0.02 and -0.01, respectively.
